# Impact of SARS-CoV-2 Pneumonia on Chronic Obstructive Pulmonary Disease: A Comparative Study in ICU Patients

**DOI:** 10.3390/v17121594

**Published:** 2025-12-09

**Authors:** Duygu Kayar Calili, Nihal Yuzbasioglu, Melih Gaffar Gozukara, Demet Bolukbasi, Isil Ozkocak Turan, Seval Izdes

**Affiliations:** 1 Department of Anesthesiology and Reanimation-Intensive Care, Faculty of Medicine, Ankara Yıldırım Beyazıt University, Ankara Bilkent City Hospital, Ankara 06800, Turkey; sevalizdes@aybu.edu.tr; 2 Department of Intensive Care, Adıyaman Training and Research Hospital, Adıyaman 02100, Turkey; nihal.yuzbasioglu@gmail.com; 3 Department of Public Health, Faculty of Medicine, Ankara Yıldırım Beyazıt University, Ankara 06800, Turkey; mggozukara@aybu.edu.tr; 4 Department of Intensive Care, Ankara Bilkent City Hospital, Ankara 06800, Turkey; de.emeet@hotmail.com; 5 Department of Anesthesiology and Reanimation-Intensive Care, University of Health Sciences, Ankara Bilkent City Hospital, Ankara 06800, Turkey; isil_ozkocak@yahoo.com

**Keywords:** chronic obstructive pulmonary disease, SARS-CoV-2 infection, intensive care, pneumonias

## Abstract

Chronic obstructive pulmonary disease (COPD) is a recognized risk factor for poor outcomes in SARS-CoV-2 infection, yet its specific impact on critically ill patients remains unclear. We aimed to compare the clinical and laboratory profiles of ICU SARS-CoV-2 pneumonia patients with or without pre-existing COPD and identify factors associated with mortality among those with COPD. In this retrospective study, adult intensive care unit (ICU) admissions for SARS-CoV-2 pneumonia (n = 1536) were divided into a COPD group (n = 253) and a non-pulmonary-disease (NPD) group (n = 1283). Demographics and clinical characteristics, severity of disease, length of stay, laboratory values, and survival outcomes were compared. COPD patients were older, had higher Acute Physiology and Chronic Health Evaluation score, and had a greater prevalence of comorbidities (*p* < 0.05). They required invasive mechanical ventilation (IMV) more frequently, had experienced higher mortality, and had shorter hospital stays (*p* < 0.05). Ferritin levels were lower in COPD patients (*p* < 0.001). Multivariate regression analysis also identified that length of hospital stay, IMV, elevated procalcitonin, and neutrophil-to-lymphocyte ratio (NLR) were associated with COPD patients’ mortality (*p* < 0.05). COPD is associated with an increased disease burden and mortality rate in critically ill SARS-CoV-2 patients. High NLR levels and IMV are significantly associated with mortality in these patients.

## 1. Introduction

SARS-CoV-2 infection can present with a wide range of clinical manifestations, from mild respiratory symptoms to severe pneumonia and critical illness requiring admission to an intensive care unit (ICU) [1]. Throughout the pandemic, various factors influencing clinical outcomes and prognosis have been investigated, particularly the impact of comorbidities and the prognostic value of different laboratory markers [1,2].

As with other viral infections, SARS-CoV-2 infection can trigger exacerbations of COPD [3]. Furthermore, a higher prevalence of COPD has been observed in patients with a more severe SARS-CoV-2 infection [4]. However, studies investigating the severity and progression of SARS-CoV-2 infection in COPD patients have produced conflicting results. While some studies suggest that SARS-CoV-2 infection is associated with greater severity and a poorer prognosis in COPD patients, others report outcomes similar to those observed in the general population [4,5,6]. Therefore, it is necessary to conduct comparative studies that specifically examine the severity of the disease and survival rates in critically ill COPD patients with SARS-CoV-2. The primary aim of this study was to compare laboratory values, disease severity, length of stay in the ICU and hospital, and survival in patients with and without a diagnosis of COPD who were being treated in the ICU due to SARS-CoV-2 infection. The secondary objective was to identify mortality-related factors in SARS-CoV-2-infected COPD patients.

## 2. Materials and Methods

### 2.1. Patients

This single-center study was conducted in the ICU of a tertiary care hospital in Ankara, Türkiye. Following obtaining approval from the hospital’s ethics committee (Ethical approval number: E2-21-58, approved on 27 January 2021), patients diagnosed with SARS-CoV-2 pneumonia and admitted to the ICU between March 2020 and January 2021 were included in the study retrospectively. Admission to the ICU was determined by the presence of significant respiratory distress or the fulfillment of any one of the following criteria: (i) respiratory compromise, defined as tachypnea (respiratory rate > 30 breaths/min), SpO_2_ < 90% or PaO_2_ < 70 mmHg despite 5 L/min oxygen therapy, or a PaO_2_/FiO_2_ ratio < 300; or (ii) systemic compromise, characterized by the presence of sepsis, septic shock, or signs of multiple organ dysfunction. The patients included in the study were categorized into two groups: those without any pulmonary disease (the non-pulmonary disease, NPD group), and those with COPD (the COPD group).

### 2.2. Clinical Assessment and Data Collection Instruments

We compared the following demographic and clinical characteristics: age; sex; comorbidities, APACHE II (Acute Physiology and Chronic Health Evaluation) score, duration of symptoms (the time between symptom onset and ICU admission), length of stay (LOS) in the ICU and hospital disease severity (categorized as moderate, severe, or critical; classified according to the World Health Organization’s classification of disease severity) [7]. Patients without pneumonia or hypoxia were classified as having mild disease. Those with clinical signs of pneumonia but with an oxygen saturation level above 90% on room air were classified as having moderate disease. Patients with clinical signs of pneumonia accompanied by at least one criterion of severe respiratory distress (respiratory rate > 30 breaths/min or oxygen saturation < 90% on room air) were classified as having severe disease. Patients with acute respiratory distress syndrome, sepsis, or septic shock were classified as having critical disease [7]. All patients admitted to the ICU had pneumonia and hypoxemia; therefore, there were no patients in the mild disease category in our ICU cohort. Modality of respiratory treatment (non-invasive or invasive; oxygen therapy via mask, high-flow nasal oxygen therapy, and non-invasive mechanical ventilation (MV) were included in the non-invasive group, while invasive MV-IMV was included in the invasive group), and survival outcomes (discharge or death) were also compared. The patients’ respiratory treatment modalities reflect the highest level of respiratory support required by the patient at any point during their entire ICU stay.

We also compared various laboratory parameters at ICU admission, such as eosinophil count (×10^9^/L), lymphocyte (%), platelet count (×10^9^/L), neutrophil-to-lymphocyte ratio (NLR), C-reactive protein (CRP mg/L), procalcitonin (PCT µg/L), D-dimer (mg/L), interleukin-6 (IL-6 pg/mL), and ferritin (µg/L) levels between the groups.

In addition to comparing the COPD and NPD groups, we compared survivor and non-survivor COPD patients based on the same variables to evaluate the factors associated with mortality.

### 2.3. Statistical Analyses

A statistical analysis was performed using SPSS version 26.0 (Armonk, NY, USA: IBM Corp.). The distribution of the data was examined using visual (histogram) and analytical (Kolmogorov–Smirnov) methods and revealing that the analyzed variables were distributed both parametrically and non-parametrically. When presenting the data, we used numbers (n), percentages (%), and also, medians (minimum–maximum/25th–75th percentile). The Pearson chi-square test was used for the statistical analysis of categorical data, and the column proportion test was used for categorical variables exceeding four. For the non-parametric comparisons of numerical data between two groups, we used the Mann–Whitney U test and, for parametric comparisons, Student’s t-test. For more than two groups, we used the one-way ANOVA for parametric data distribution. The non-parametric comparison of more than two groups was performed using the Kolmogorov–Smirnov test. We applied Bonferroni correction to the post hoc tests. Binomial regression was used to determine the factors affecting mortality. When creating the model, we only used variables that had a statistical difference in relation to the outcome of death. In the regression model evaluating factors associated with mortality, although the lymphocyte count reached statistical significance in the pairwise comparisons, it was intentionally excluded to mitigate potential issues of multicollinearity and redundancy, as NLR, which incorporates lymphocyte dynamics, was already included as a primary inflammatory biomarker. To avoid multicollinearity, we tested the model structure with each factor present and absent and performed a collinearity statistics test. The minimum tolerance of all the variables in the regression model was found to be 0.810. We accepted statistical significance at *p* < 0.05 with a 95% confidence interval.

## 3. Results

A total of 1592 patients were diagnosed with SARS-CoV-2 pneumonia and admitted to the ICU during the study period. Patients with pulmonary disease other than COPD (n = 36) and those with missing data (n = 20) were excluded from the study. Thus, the remaining 1536 patients were divided into two groups: NPD (n = 1283) and COPD (n = 253).

A comparison of the patients’ characteristics is presented in Table 1. Significant differences were observed between groups in terms of age, the presence of hypertension (HT), cardiovascular disease (CVD) and neurological disease (ND), APACHE II scores, length of hospital stay, respiratory treatment modalities, and survival outcomes. (*p* < 0.05) (Table 1). When compared to the NPD group, patients in the COPD group were older, had a higher prevalence of HT and CVD comorbidities, as well as a lower prevalence of ND (*p* < 0.05). Additionally, the COPD group also had higher APACHE II scores, a greater need for IMV, and higher mortality rates, as well as shorter hospital stays, compared to the other group (*p* < 0.05). No statistically significant differences were observed between the groups regarding sex, symptom duration, length of ICU stay, or disease severity (*p* > 0.05).

When the groups were compared laboratory parameters, ferritin levels were significantly lower in COPD patients (*p* < 0.001). However, no significant differences were observed in the levels of eosinophils, lymphocytes, platelet counts, NLR, CRP, PCT, D-dimer, or IL-6 levels (*p* > 0.05) (Table 2).

Comparisons of the characteristics between COPD patients who were discharged and those who died in the ICU revealed that among COPD patients, those who died had higher APACHE II scores and IMV rates, as well as a shorter length of hospital stay than those who survived (*p* < 0.05) (Table 3). However, no significant differences were found in terms of gender, comorbidity burden, symptom duration, and length of stay in the ICU when comparing COPD patients who died versus those who survived (*p* > 0.05). Since there were no non-survivor patients in the ‘moderate’ disease severity group, the *p*-value could not be calculated.

Compared to survivors, non-survivors with COPD had lower counts of eosinophils, lymphocytes, and platelets, while their NLR, PCT, and IL-6 levels were higher (*p* < 0.05) (Table 4). There were no significant differences in CRP, D-dimer, and ferritin levels between the two groups (*p* > 0.05).

A regression analysis of parameters that showed significant differences between SARS-CoV-2-positive survivor and non-survivor COPD patients revealed that length of hospital stay, IMV, NLR, and PCT were independently associated with mortality in COPD patients (*p* < 0.05) (Table 5).

## 4. Discussion

In our study, the severity of SARS-CoV disease at admission to the ICU was similar in patients with COPD and those without COPD. However, we found that patients with SARS-CoV-2 and COPD were older, had higher APACHE II scores, and a higher prevalence of comorbidities such as HT and CVD. Additionally, the COPD group had higher IMV rates, increased mortality, shorter hospital stays, and lower ferritin levels at admission. When overall factors affecting the mortality of COPD patients with SARS-CoV-2 were evaluated, shorter length of hospital stay and higher rates of IMV were found to be significantly associated with mortality. Additionally, higher levels of PCT and NLR also demonstrated a significant association with COPD mortality.

In fact, advanced age has been reported as one of the risk factors for mortality among hospitalized SARS-CoV-2 patients [1,8]. The prevalence of COPD is also known to rise with age, particularly in individuals over 60 years old [9]. Our findings are partially consistent with this general trend: our COPD cohort was significantly older than the non-COPD group, aligning with the observation that older individuals are more susceptible to both COPD and severe COVID-19. However, in our specific analysis of survival within the COPD group, we found that discharged and deceased COPD patients were similar in age. The groups in our study were also similar in terms of gender. These findings contrast with the study from the United States by Puebla Neira et al., which reported that mortality was higher among female COPD patients and in those aged 40–79 years [10]. The absence of an age-related difference in COPD mortality in our study may reflect the naturally high-risk profile of critically ill older patients admitted to the ICU with SARS-CoV-2 infection, where other factors, such as physiological status, may play a more dominant role in predicting outcomes.

Regarding comorbidities, our study supports the international literature: comorbidities such as HT and CVD were observed at a higher prevalence in our COPD cohort compared to the NPD group, a pattern frequently reported in studies comparing these patient populations [10,11,12]. A study of 336 SARS-CoV-2 patients from China found that the prevalence of CVD and ND was higher in COPD patients than in those without COPD [11]. Andreen and colleagues in their study from Sweden of patients infected with SARS-CoV-2 reported that comorbidities, particularly CVD, were more prevalent in COPD patients than in those without COPD [12]. They also highlighted that the presence of CVD, alongside a higher Charlson Comorbidity Index (CCI) score, was associated with increased mortality in that group [12]. Similarly, a Spanish study by Antúnez et al. found that COPD patients generally had higher CCI scores, reflecting a greater burden of comorbidity and a subsequently higher mortality rate [6]. Our result, which shows a higher comorbidity burden in COPD patients, is consistent with global observations, confirming that comorbidities contribute significantly to the overall susceptibility of this group during critical SARS-CoV-2 infection. We believe that chronic inflammation and low physical activity due to respiratory distress may contribute to the higher prevalence of these comorbidities in COPD patients. However, the prevalence of ND in COPD patients was unexpectedly lower in our study than in patients without COPD. This finding contradicts previous reports suggesting that chronic respiratory disease is frequently associated with neurological comorbidities, particularly in elderly populations [6,13,14]. During the pandemic, patients with advanced COPD and severe neurological disorders may not have been able to seek hospital care due to severely limited physiological reserves, meaning they may have been underrepresented among our ICU admissions. Furthermore, we believe that our findings may partially reflect the limitations of retrospective data collection. Although HT and CVD were more prevalent in COPD patients in our study, we found that these comorbidities were not associated with mortality in this group.

Previous studies have reported conflicting results regarding the impact of COPD on the severity and prognosis of SARS-CoV-2. The literature is divided, with some reports identifying COPD as a risk factor for severe disease, citing increased rates of ICU admission and the need for MV [10,11,15,16]. Conversely, other large-scale studies have found no significant difference in prognosis when comparing COPD patients to the general population [4,5,17,18]. This discrepancy may complicate efforts to separate the role of COPD from that of other confounding variables. It has been suggested that increased angiotensin-converting enzyme 2 (ACE2) expression in the airways of COPD patients may contribute to worse outcomes [3]. A study by Liu et al. from the United States, evaluating SARS-CoV-2 infected patients with and without airway diseases, observed the highest Sequential Organ Failure Assessment (SOFA) scores and mortality rates in COPD patients [15]. Our study contributes to the findings suggesting a worse trajectory for the COPD patients. While we found no significant difference in the categorized disease severity between COPD and NPD groups, our data revealed higher APACHE II scores, greater requirement for IMV, and increased mortality rates in COPD patients. Non-survivors with COPD had higher APACHE II scores and shorter hospital stays than survivors. Although we could not assess it statistically, we believe that the overwhelming majority of non-survivor patients having critical disease severity is clinically significant. This result suggests that the non-survivor group presented with a substantially higher burden of severe and critical disease at hospital admission. However, in multivariate analysis, we could not establish a correlation between APACHE II scores and mortality in COPD patients. These findings may indicate that COPD patients may experience faster and more severe clinical progression of SARS-CoV-2 pneumonia and worse outcomes due to their weakened respiratory systems, and the progression to a critical state represents the crucial, irreversible tipping point towards mortality in this patient population.

Intubation and IMV are crucial for patients with SARS-CoV-2 pneumonia complicated by severe oxygenation impairment. Beyond being merely a therapeutic intervention, the necessity for IMV serves as a robust clinical marker of the most severe form of the disease, directly signifying the failure of less-invasive support and the presence of critical respiratory failure [4,8,11,19]. Consistent with global observations, the IMV rate has been reported to be significantly higher in COPD patients with SARS-CoV-2 infection, such as in a study by Attaway et al. from the United States [17]. The decision to proceed with IMV may be complex, as this intervention carries a significant risk of complications and is frequently associated with increased mortality, particularly in this vulnerable patient population [19,20]. One of the important findings of our study is the prominence of non-invasive respiratory treatment strategies among discharged COPD patients.

In contrast, the increased rates of IMV and mortality observed in our COPD cohort may suggest that underlying respiratory dysfunction significantly exacerbates the clinical trajectory in this population. This observation may reinforce the critical vulnerability of COPD patients, where pre-existing respiratory compromise leads to a greater requirement for advanced respiratory support. Furthermore, the higher frequency of IMV among our non-survivor COPD patients, coupled with the finding that this method was independently and significantly associated with mortality in the multivariate analysis, may also suggest that the failure of non-invasive strategies and progression to IMV indicate a crucial stage in disease progression. It must be acknowledged that the inclusion of IMV treatment and length of hospital stay in the multivariate model presents a methodological limitation. As these are post-baseline variables, the model’s results should be interpreted as factors associated with mortality during the critical care course, rather than prognostic factors. We also note that while the strong association between IMV use and mortality is clinically robust, the resulting extreme Odds Ratio and wide Confidence Interval suggest a statistical phenomenon known as complete separation. This issue limits the precise quantitative interpretation of IMV’s effect size within our multivariate framework, despite its clear clinical relevance. A short length of hospital stay should not be considered a factor influencing or predicting mortality; rather, it should be interpreted as a variable closely associated with mortality and even as a reflection of clinical deterioration. In our study, the shorter length of hospital stay observed in COPD patients is likely attributable to their higher mortality rather than faster recovery. These patients may be less resilient to the acute stress of severe SARS-CoV-2 pneumonia. Consequently, they may have died earlier in the ICU, resulting in shorter stays. We acknowledge that the severity of symptoms in COPD patients can vary, but we did not have information on the COPD stage in our cohort. We believe that future comparisons stratified by COPD stage would provide more granular and clinically meaningful insights into the effectiveness of non-invasive respiratory treatment strategies.

Hematological changes, including lymphopenia, eosinopenia, and neutrophilia, alongside increased NLR, CRP, IL-6, ferritin, and D-dimer levels, have been consistently associated with disease prognosis and mortality in SARS-CoV-2 infection across various geographical regions [1,2,21,22]. Similarly, patients with COPD often exhibit higher levels of leukocytes, neutrophils, D-dimer, CRP, ferritin, and PCT, and lower lymphocyte counts compared to those without COPD [4,11,23]. Furthermore, in a study from China, Chen et al. found that low lymphocyte counts and elevated IL-6 levels were associated with disease severity in patients with SARS-CoV-2 infection and chronic airway disease [24]. He et al. in China reported higher PCT levels in patients with COPD and SARS-CoV-2 infection [11]. In our study, the only observed difference in laboratory values when comparing the COPD and NPD patients was lower ferritin levels in the COPD cohort. The similarity in laboratory values between the COPD and non-COPD groups suggests that, even in the presence of an underlying chronic inflammatory state in patients with COPD, both groups may have exhibited comparable responses to the acute inflammation induced by SARS-CoV-2 infection.

However, non-survivor COPD patients showed lower eosinophil, lymphocyte, and platelet counts, and higher NLR, PCT, and IL-6 levels than survivors. Within these parameters, only PCT and NLR were independently associated with mortality. Given that PCT is widely regarded as a marker of bacterial superinfection and sepsis, its independent association in our model may indicate that secondary bacterial complications contribute to mortality in this vulnerable cohort, in addition to the underlying viral pathology. Patients with COPD are predisposed to bacterial infections, and assessing PCT levels may be important for guiding the initiation of antimicrobial therapy and planning supportive care. The elevated IL-6 levels observed in the non-survivor group may be related to the severe inflammatory state in this group. Although platelet levels might be expected to be higher in patients with COPD compared to those without COPD [17], a decrease in platelet counts may be observed in the setting of severe SARS-CoV-2 infection [1,2]. Collectively, these findings suggest that mortality in COPD patients is preceded by a state of severe, uncontrolled inflammation, potential secondary infection, and profound hematological stress.

Ferritin has been reported as a biomarker associated with SARS-CoV-2 infection severity in hospitalized patients [21,22,25]. In our study, although COVID-19 severity was similar between COPD and non-COPD groups, ferritin levels were significantly lower in COPD patients. Crucially, despite the elevated mean levels in both groups, ferritin did not demonstrate a significant difference between survivors and non-survivors within the COPD cohort. Consequently, ferritin was not found to be independently associated with mortality in our multivariate analysis. However, the relatively lower ferritin levels in COPD patients may warrant further investigation, given that COPD severity is associated with iron deficiency and low ferritin levels are linked to airflow limitation and smoking [26]. Non-anemic iron deficiency (NAID), characterized by low iron/ferritin, has been identified as a predictor of exacerbations and hospitalizations in COPD patients [27]. Furthermore, new evidence suggests that abnormal iron metabolism and ferroptosis (iron-dependent cell death) play a role in COPD pathogenesis, potentially resulting from unstable iron accumulation in lung epithelial cells exposed to cigarette smoke and subsequent increase in lipid peroxidation [28,29]. Thus, the relatively lower ferritin levels observed in COPD patients with SARS-CoV-2 infection in our study may be related to ferroptosis. However, this topic should be further explored in future studies.

NLR has been linked to disease severity and mortality in numerous SARS-CoV-2 studies [21,22]. In our study, NLR was independently associated with mortality. Our findings suggest that the underlying chronic inflammatory state in COPD may contribute to a higher systemic inflammatory burden, which is further aggravated by SARS-CoV-2 infection, potentially leading to a more severe disease course. Therefore, NLR may represent a practical and easily accessible biomarker for risk stratification in this population.

Eosinopenia may indicate increased systemic inflammation or impaired immunity during viral infections [30], and it has been associated with disease progression and mortality in SARS-CoV-2 infection [4,5,31]. Eosinophils are known to produce ribonuclease proteins that degrade viral RNA [32,33]. Consistent with the literature, Chen et al. [24] in China and Liu et al. [15] in the United States found that low eosinophil counts were associated with more severe disease or mortality, irrespective of chronic airway disease. In our study, eosinophil levels were similar between the COPD and NPD groups, and no association was found between eosinophil levels and mortality in multivariate analysis. We suggest that the widespread use of corticosteroids in both groups may have influenced these results, as corticosteroids are known to affect complete blood count parameters [34,35].

Our study presents descriptive comparisons and analytical results concerning the risk factors associated with mortality in patients with COPD and SARS-CoV-2 infection. The statistical power and generalizability of the findings are increased by utilizing a large sample size. While this study provides important information for critical care management by focusing on intensive care patients, it also has some limitations. Firstly, the retrospective, single-center design may limit its generalizability. Secondly, the effect of medications on laboratory parameters was not examined. Thirdly, our results may have been affected by the fact that we did not specifically examine the types or severity of COPD. Patient enrollment in our study relied on pre-existing medical records, which may introduce the risk of diagnostic inaccuracy. We lacked detailed objective data, including spirometry results, smoking history, and imaging findings, which are essential for rigorous characterization and staging of COPD. Furthermore, relying on established diagnoses means that patients with undiagnosed or subclinical COPD may have been inadvertently categorized into the NPD group. This misclassification bias could potentially alter the true differences in laboratory parameters and outcomes observed between groups.

## 5. Conclusions

In conclusion, SARS-CoV-2-infected COPD patients in the ICU were older, had a higher prevalence of comorbidities, and required more IMV compared to the NPD patients. They also had shorter hospital stays due to higher mortality rates. Additionally, high PCT, NLR, and IMV were associated with mortality in COPD patients. PCT and NLR are easily accessible parameters that may serve as valuable indicators of increased disease severity and potential for clinical deterioration, warranting rapid and aggressive supportive management in this vulnerable subgroup. Although ferritin was not related to mortality, the lower ferritin levels in COPD patients—despite similar overall disease severity compared to NPD patients—may reflect underlying pathogenetic mechanisms such as ferroptosis. This situation could lead to misdiagnosis in SARS-CoV-2-infected COPD patients in the ICU if disease severity is determined based on ferritin levels alone. To clarify these relationships, prospective studies incorporating COPD staging, longitudinal biomarker monitoring, and treatment history are required.

## Figures and Tables

**Table 1:** Comparison of the groups’ characteristics.

		NPD Group (n = 1283)	COPD Group (n = 253)	*p* Value
Age (years)		68.84 ± 14.83	74.72 ± 9.95	<0.001 ^a^
Sex (n/%)	Female	505 (39.4%)	103 (40.7%)	
	Male	778 (60.6%)	150 (59.3%)	0.688 ^b^
Present comorbidities (n/%)	HT	568 (44.3%)	149 (58.9%)	<0.001 ^b^
	CVD	353 (27.5%)	112 (44.3%)	<0.001 ^b^
	CKD	116 (9.0%)	17 (6.7%)	0.230 ^b^
	DM	382 (29.8%)	79 (31.2%)	0.645 ^b^
	ND	239 (18.6%)	27 (10.7%)	0.002 ^b^
	Malignancy	120 (9.4%)	23 (9.1%)	0.896 ^b^
APACHE II		19.22 ± 9.13	21.42 ± 8.81	0.001 ^a^
Duration of symptoms (days)		7.05 ± 3.28	7.22 ± 3.44	0.453 ^a^
Length of ICU stay (days)		10.78 ± 7.94	10.35 ± 8.52	0.213 ^c^
Length of hospital stay (days)		15.00 ± 9.05	14.18 ± 9.65	0.041 ^c^
Severity of disease (n/%)	Moderate	60 (4.7%)	13 (5.1%)	
	Severe	174 (13.6%)	29 (11.5%)	0.648 ^b^
	Critical	1049 (81.8%)	211 (83.4%)	
Respiratory treatment Modalities (n/%)	Non-invasive	743 (57.9%)	118 (46.6%)	0.001 ^b^
	Invasive	540 (42.1%)	135 (53.4%)	
Survival (n/%)	Discharge	761 (59.3%)	125 (49.4%)	0.004 ^b^
	Death	522 (40.7%)	128 (50.6%)	

NPD: No pulmonary disease, COPD: chronic obstructive pulmonary disease, APACHE II: Acute Physiology and Chronic Health Evaluation II, ICU: intensive care unit, HT: hypertension, CVD: cardiovascular disease, CKD: chronic kidney disease, DM: diabetes mellitus, ND: Neurological disease, ^a^: Independent *T* test, ^b^: Pearson chi-square test, ^c^: Mann–Whitney U test. Data were presented (n/%) and mean ± sd.

**Table 2:** Comparison of the groups’ laboratory values.

	NPD Group (n = 1283)	COPD Group (n = 253)	*p* Value ^c^
Eosinophil (×10^9^/L)	0.49 ± 0.82	0.51 ± 0.78	0.557
Lymphocyte (%)	11.33 ± 10.97	9.76 ± 6.52	0.078
Platelet (×10^9^/L)	248.09 ± 115.35	244.68 ± 111.35	0.666
NLR	12.90 ± 12.94	14.30 ± 12.61	0.093
CRP (mg/L)	124.91 ± 100.11	122.72 ± 93.14	0.766
PCT (µg/L)	0.41 ± 0.72	0.36 ± 0.58	0.466
D dimer (mg/L)	4.99 ± 14.14	5.51 ± 17.56	0.447
IL-6 (pg/mL)	124.97 ± 212.03	139.79 ± 260.81	0.337
Ferritin (µg/L)	872.54 ± 1551.85	651.48 ± 896.26	<0.001

NPD: No pulmonary disease, COPD: chronic obstructive pulmonary disease, NLR: neutrophil-to-lymphocyte ratio, CRP: C-reactive protein, PCT: Procalcitonin, IL-6: Interleukin-6, ^c^: Mann–Whitney U test.

**Table 3:** Comparison of characteristics between COPD survivors and non-survivors.

		Survivor (n = 125)	Non-Survivor (n = 128)	*p* Value
Age (years)		74.79 ± 11.08	74.66 ± 8.73	0.914 ^a^
Sex (female)		53 (42.4%)	50 (39.1%)	0.589 ^b^
Comorbidities	HT	67 (53.6%)	82 (64.1%)	0.091 ^b^
	CVD	57 (45.6%)	55 (43.0%)	0.674 ^b^
	ND	14 (11.2%)	123 (10.2%)	0.840 ^b^
	Renal disease	5 (4%)	12 (9.4%)	0.088 ^b^
	DM	36 (28.8%)	43 (33.6%)	0.411 ^b^
	Malignancy	8 (6.4%)	15 (11.7%)	0.141 ^b^
APACHE II		15.78 ± 5.21	26.93 ± 8.09	<0.001 ^a^
Duration of symptoms (days)		7.12 ± 3.21	7.31 ± 3.65	0.724 ^a^
Length of ICU stay (days)		10.16 ± 8.38	10.54 ± 8.67	0.644 ^c^
Length of hospital stay (days)		15.62 ± 9.77	12.77 ± 9.36	0.002 ^c^
Severity of disease (n/%)	Moderate	73 (100%)	0 (0%)	
	Severe	202 (99.5%)	1 (0.5%)	n/c
	Critical	649 (51.5%)	611 (48.5%)	
Respiratory treatment modalities	Non-invasive	117 (93.6%)	1 (0.8%)	<0.001 ^b^
	IMV	8 (6.4%)	127 (99.2%)	

HT: hypertension, CVD: cardiovascular disease, ND: Neurological disease, APACHE II: Acute Physiology and Chronic Health Evaluation II, IMV: invasive mechanical ventilation, NLR: neutrophil-to-lymphocyte ratio, IL-6: interleukin-6, ^a^: Independent *T* test, ^b^: Pearson chi-square test, ^c^: Mann–Whitney U test, n/c: not calculated.

**Table 4:** Comparison of laboratory results between COPD survivors and non-survivors.

	Survivor (n = 125)	Non-Survivor (n = 128)	*p* Value ^c^
Eosinophil (×10^9^/L)	0.62 *±* 0.80	0.41 *±* 0.74	<0.001
Lymphocyte (%)	11.27 ± 6.83	8.27 ± 5.86	<0.001
Platelet (×10^9^/L)	264.54 ± 114.14	225.89 ± 105.43	0.008
NLR	10.88 ± 10.08	17.65 ± 13.91	<0.001
CRP (mg/L)	113.19 ± 80.92	132.03 ± 108.48	0.264
PCT (µg/L)	0.31 ± 0.55	0.41 ± 0.61	0.002
D dimer (mg/L)	4.39 ± 19.70	6.60 ± 15.18	0.104
IL-6 (pg/mL)	74.17 ± 111.97	203.87 ± 337.16	<0.001
Ferritin (µg/L)	570.38 ± 897.25	730.67 ± 891.66	0.174

NLR: neutrophil-to-lymphocyte ratio, CRP: C-reactive protein, PCT: Procalcitonin, IL-6: Interleukin-6, ^c^: Mann–Whitney U test.

**Table 5:** Analysis of related factors for the mortality of COPD patients.

	*p* Value	Odds Ratio	95% CI
APACHE II	0.058	-	-
Length of hospital stay	0.010	1.083	1.019–1.150
IMV	<0.001	38,908.467	117.078–12,930,435.95
Eosinophil (×10^9^/L)	0.215	-	-
Platelet (×10^9^/L)	0.100	-	-
NLR	0.011	1.134	1.028–1.250
IL-6 (pg/mL)	0.301	-	-
PCT (µg/L)	0.029	-	-

APACHE II: Acute Physiology and Chronic Health Evaluation II, IMV: invasive mechanical ventilation, NLR: neutrophil-to-lymphocyte ratio, IL-6: Interleukin-6, Hosmer-Lemeshow Test *p* value is: 0.995, Cox& Snell *R*^2^ value is 0.705, Nagelkerke *R*^2^ value is 0.940.

## Data Availability

The data presented in this study are available on reasonable request from the corresponding author due to legal and ethical restrictions. The authors do not have permission to share raw data.

## Abbreviations

The following abbreviations are used in this manuscript:

COPD	Chronic obstructive pulmonary disease
ICU	Intensive care unit
NPD	Non-pulmonary disease
APACHE	Acute Physiology and Chronic Health Evaluation
IMV	Invasive mechanical ventilation
NLR	Neutrophil-to-lymphocyte ratio
CRP	C-reactive protein
PCT	Procalcitonin
IL-6	Interleukin-6
HT	Hypertension
CVD	Cardiovascular disease
CKD	Chronic kidney disease,
DM	Diabetes mellitus
ND	Neurological disease
CCI	Charlson comorbidity index
ACE2	Angiotensin-converting enzyme 2
SOFA	Sequential Organ Failure Assessment
NAID	Non-anemic iron deficiency
